# The relationship between gender differences in dietary habits, neuroinflammation, and Alzheimer’s disease

**DOI:** 10.3389/fnagi.2024.1395825

**Published:** 2024-04-17

**Authors:** Alison Warren

**Affiliations:** The Department of Clinical Research and Leadership, George Washington University School of Medicine and Health Sciences, Washington, DC, United States

**Keywords:** Alzheimer’s disease, dementia, gender, sex, diet, Mediterranean diet, MIND diet, habit

## Abstract

Neurocognitive decline is one of the foremost dire issues in medicine today. The mechanisms by which dementia pathogenesis ensues are complicated and multifactorial, particularly in the case of Alzheimer’s disease (AD). One irrefutable, yet unexplained factor is the gender disparity in AD, in which women are disproportionately affected by AD, both in the rate and severity of the disease. Examining the multifaceted contributing causes along with unique gender dynamics in modifiable risk factors, such as diet, may lend some insight into why this disparity exists and potential paths forward. The aim of this brief narrative review is to summarize the current literature of gender differences in dietary habits and how they may relate to neuroinflammatory states that contribute to AD pathogenesis. As such, the interplay between diet, hormones, and inflammation will be discussed, along with potential interventions to inform care practices.

## 1 Introduction

Gender dynamics have been a ubiquitous element in human thought and behavior throughout our evolutionary process. They interact with genetics, environment, and culture to influence a myriad of daily choices that ultimately affect our overall lifestyle and related state of health. At the intersection of gender and dietary habits lies a multitude of intrapersonal (e.g., biological, psychological) and interpersonal (e.g., sociocultural, socioeconomic, cultural) factors (Grzymislawska et al., 2020). The nuances of gender differences in dietary habits continue to be explored in efforts to inform clinicians and the public about optimal nutrition for optimal health. The aim of this brief narrative review is to explore the current literature and highlight potential of gender-specific dietary habits and their impact on neuroinflammatory states that may contribute to AD pathogenesis. Neuronutrition, in this regard, entails the study and exploration of nutrients, diet, eating behavior, and food environments and their interplay with epigenetic, immune, metabolic, and psychosocial constructs in the development of neurological disorders (Badaeva et al., 2023). This review further aims to include the additional level of gender considerations in neuronutrition, which are important factors to consider in dietary advice during clinical encounters and dietary guidelines for the public. A personalized approach that considers the current evidence of gender-related differences in nutrition, metabolism, and neurocognitive risk is imperative to optimize clinical advice, yet sufficient information is still lacking to inform clinicians. While acknowledging the need for more advanced research, these aims are motivated by the endeavor to explore the research questions, (1) “how and to what extent do dietary habits differ between genders?” and (2) “how and to what extent might those gender differences in dietary habits contribute to inflammatory processes related to AD development?”

The differences between biological sex and cultural gender are important to recognize at this juncture. For the purposes of this paper, the terms “sex” and “gender” will be used interchangeably to denote one’s biological sex at birth with associated psychosocial factors, as both interact to construct definitions of “male” and “female” (Ferretti et al., 2021) and influence habits and disease risk.

### 1.1 Gender differences in dietary habits

#### 1.1.1 Eating behaviors

Eating behavior is influenced by a myriad of factors that go beyond the basic metabolic needs of the individual, such as food type preferences (Grzymislawska et al., 2020); attitudes and beliefs about food type (Modlinska et al., 2020; Rosenfeld and Tomiyama, 2021); religious practices (Gilbert and Khokhar, 2008); sociocultural factors (Jimenez-Morcillo and Clemente-Suárez, 2023); body image perceptions (Boraita et al., 2020; Jimenez-Morcillo and Clemente-Suárez, 2023), and other external influences such as social media (Jacob and Panwar, 2023). In the case of females especially, there appears to be a relationship between quality of life and body satisfaction, and a higher propensity toward disordered eating (Boraita et al., 2020). Additionally, energy homeostasis may be altered by physiological abnormalities that affect appetite and caloric needs, such as metabolic and endocrine disorders (Rogowicz-Frontczak et al., 2017). Ideally, appetite and food intake are regulated by a balance between leptin, the satiety-promoting hormone, and ghrelin, the main hunger-promoting hormone (orexigenic) (Egmond et al., 2023). Physiological differences between genders that can affect appetite may also be present that are not pathological in nature, such as sex differences in the impact ghrelin on food intake. Leone et al. (2022) measured ghrelin in 24 healthy, normal weight volunteers before and after a balanced meal. No sex differences were found between appetite ratings and ghrelin levels in the fasting state, suggesting a normal physiological response in both sexes. However, postprandially, women tended to attain satiety earlier than men, who instead reached the nadir of hunger later, both of which corresponded to findings of greater ghrelin suppression in women compared to men (Leone et al., 2022).

The balance between energy needs and expenditure can also influence dietary habits and based on differing body compositions, may differ between genders. Bi et al. (2019) conducted a cross-sectional study of 261 male and female participants to examine this relationship between energy metabolism and gender. They found that lean body mass was positively correlated with protein and fat intake in females but not males. No significant relationships were found between fat mass and macronutrient intake in males or females (Bi et al., 2019). Other factors such as sleep loss can alter the balance between ghrelin, leptin, and adiponectin (another appetite-regulating hormone), and may affect the metabolic response differently between genders, but thus far, the observed sex differences in metabolic response to sleep loss (i.e., increased hunger and weight gain) have been inconsistent and require further investigation (Egmond et al., 2023).

Eating behaviors are influenced by factors that exceed the basic homeostatic needs of the organism, such as eating for pleasure, desire, social influences, and based on emotional influences. These “non-homeostatic determinants” of food intake, such as food cravings, affect individuals differently and can contribute to obesity along with associated comorbid diseases when cravings lead to overconsumption of sugary and fat-rich snack foods (Richard et al., 2017, p. 215). Food cravings, therefore, also serve as an influencing factor in dietary intake. While both genders experience food cravings, the underlying mechanisms may differ. For example, a study by Klimesova et al. (2020) surveyed 1,394 participants and found that the intensity of cravings did not differ between males and females, but males associated cravings with positive outcome expectations (i.e., positive or negative reinforcement), whereas females scored higher for emotional cravings (Klimesova et al., 2020). When food cravings regularly result in the increased consumption of unhealthy foods, especially ultra-processed foods associated with the Western diet that are pro-inflammatory, it can lead to inflammageing—systemic inflammation and neuroinflammation that may increase the risk for chronic diseases, including AD (Więckowska-Gacek et al., 2021).

#### 1.1.2 Food preferences

Dietary habits are further influenced by the likes and preferences of the individual. Food preferences naturally vary among individuals, but some trends have been suggested between genders. A recent study by Feraco et al. (2024) utilized an online survey administered to 2,198 participants in Italy to investigate gender differences in food preferences and eating habits. Their results suggest that men prefer and consume more red and processed meat; women are more inclined toward healthier foods (e.g., vegetables, whole grains, tofu, high-cocoa dark chocolate); men tend to skip snacks while women tend to eat more frequently, report higher hunger levels in the morning, and more frequent episodes of uncontrolled eating without hunger; men are more likely to eat alone, eat quickly, and dine out. Their findings contribute important information for personalized nutrition recommendations and health promotion strategies based on gender dynamics (Feraco et al., 2024).

Another recent review by Grzymislawska et al. (2020) identified several gender differences in nutritional behaviors and dietary habits. They found that women gravitate toward healthy nutrition, deliberate control of body weight, an inclination toward eating in groups and in stressful situations, and exhibit frustration surrounding their own eating habits reflecting social influence and a negative perspective and tendency toward reducing eating for pleasure; whereas men prefer meals with high fat content and strong taste and strongly motivated by eating for pleasure, and more frequently dine at fast food restaurants, use dietary supplements, and secretly eat sweet foods while watching television (Grzymislawska et al., 2020). A similar cross-sectional study by Lombardo et al. (2019) examined gender differences in food habits in 2,021 adults. They found that women consumed more whole grains, cereals, vegetables, water, sugar-sweetened beverages, and alcoholic drinks; and were more likely to eat uncontrollably, missed more meals and ate more frequently during the day compared to males. Men were found to consume more eggs, meat, processed meat; and were more likely to eat during the night, eat meals out more often, and tended to be hungrier later in the day compared to females (Lombardo et al., 2019).

Variations in psychological personality traits may be a further source of dietary choices, habits, and preferences, and potentially add another layer of depth to gender differences in nutrition. In this vein, an interesting study by Jimenez-Morcillo and Clemente-Suárez (2023) examined, among other things, physical activity, gender differences in dietary patterns along with psychological traits. From their sample of 605 strength-training participants, they found that, similar to other studies, females prefer healthier options such as vegetables and consume more water, whereas males prefer milk, fermented products, carbohydrates and alcoholic and carbonated beverages. Further, they found that males scored higher for extraversion, and females demonstrated higher conscientiousness, openness, and more negative thoughts and anxiety (Jimenez-Morcillo and Clemente-Suárez, 2023). These findings provide further insight into gender differences and lifestyle choices.

#### 1.1.3 Attitudes and beliefs

Gender differences in food intake and preferences are also shaped by attitudes and beliefs, both culturally and individually. Environmental issues notwithstanding, a link between red and processed meat consumption has been associated with increased mortality risk from diseases such as cardiovascular disease and cancer (Weber and Kollmayer, 2022). The literature has supported a striking difference between the sexes regarding meat and plant consumption. The meat-based diet is dominant in Western culture, and men consume more meat than women across Europe, Asia, Africa, and the Americas (Modlinska et al., 2020). In fact, evidence has consistently showed that men are more attached to meat consumption, more resistant to and defensive toward plant-based diets (Hinrichs et al., 2022). Women are twice as likely to follow a vegetarian diet even across Western societies (Modlinska et al., 2020). The results of a systematic review of 29 articles by Modlinska et al. (2020) found that men tend to have more positive attitudes toward meat, which is commonly perceived to be a “masculine” food, whereas dairy products, fruit and vegetables are more commonly considered “feminine” foods. Their findings also revealed that this gender-bias is further evidenced by the tendency for omnivores to exhibit more prejudice against vegetarian men than vegetarian women (Modlinska et al., 2020). Another study by Rosenfeld and Tomiyama (2021) examined the relationship between gender role conformity and gender identity for meat consumption and openness to becoming vegetarian. They found that greater conformity to traditional gender roles predicted greater consumption of beef and chicken and lower openness to vegetarianism in men, but no relationship was found in women, and no effects were observed for pork or fish. Greater traditional gender role conformity and gender identity were associated with openness to vegetarianism for health reasons in women, and lower traditional gender role conformity was associated with openness to vegetarianism for environmental reasons in men (Rosenfeld and Tomiyama, 2021). A study by Hinrichs et al. (2022) further explored the meat-masculinity link utilizing a survey that measured various expressions of defensiveness toward plant-based eating after participants watched a video promoting plant-based diets. They found that males scored higher than females in all measures of defensiveness, and negative affect toward plant-based eating mediated the associations between gender and defensiveness (Hinrichs et al., 2022). Finally, Ritzel and Mann (2021) investigated whether this gender bias toward meat consumption was stable across stages of human life. Their results suggest that gender differences in meat consumption emerge after the age of four, move in parallel with biological development, and reach a maximum between 51 and 65 years of age.

### 1.2 Gender considerations in Alzheimer’s disease

#### 1.2.1 AD and inflammation

AD is characterized by progressive impairments in several cognitive domains, including memory, executive function, language, mood, and activities of daily living (Alzheimer’s Association, 2023) as the result of neuronal loss, extracellular amyloid-beta accumulation and intracellular tau containing neurofibrillary tangles in the brain (Busche and Hyman, 2020). AD and other neurological disorders also involve inflammation in the central nervous system (CNS), otherwise known as “neuroinflammation”, which can occur as a result of numerous factors, such as aging, autoimmunity, injury, infection, and systemic inflammation (Mukhara et al., 2020), to name a few. Proinflammatory cytokines (e.g., TNF-, IL-1, and IL-6) are upregulated in the brains of persons with AD, suggesting inflammation is a hallmark contributor to AD development (Sinyor et al., 2020). Although protective in the short-term, chronic neuroinflammation is the product of a complex symphony of overactive microglia and astrocytes that produce proinflammatory cytokines a dysregulated immune response in the CNS and periphery, hormone dysregulation (including sex hormones), and alterations in the microbiome (i.e., dysbiosis) (Scassellati et al., 2021; Saha et al., 2024). AD pathogenesis is a multifactorial process in the CNS as well as the periphery in this regard, the hallmarks of which appear several decades before the onset of clinical symptoms (Pistollato et al., 2018). Although only one piece of the puzzle, eating habits may contribute to the systemic inflammatory and neuroinflammatory cascade described above, and may therefore also serve as an early prevention strategy.

An unhealthy diet consisting of added sugars, chemicals, pro-inflammatory fats and oils, and ultra-processing methods serve as a major driver of inflammatory processes in the body. The Western diet in particular has been associated with an increased risk of AD development (Jena et al., 2018; Więckowska-Gacek et al., 2021). High intake of meat, especially red meat, seen in developed countries contributes to copper absorption and has been linked to dementia and AD (Pistollato et al., 2018). It is likely unsurprising that diets low in fiber and high in sugar, refined carbohydrates, saturated and trans-fats, and alcohol are associated with increased risk of AD also concomitantly result in negative effects on the microbiome (Frausto et al., 2021). Owing to an induction of sustained metabolic, immune, and microbiome alterations, systemic inflammation drives neuroinflammation via blood brain barrier (BBB) breakdown, amyloid accumulation, synaptic dysfunction, and neurodegeneration that ultimately triggers AD (Jena et al., 2018; Więckowska-Gacek et al., 2021).

Moreover, changes in dietary consumption occur in both genders with aging as well as neurodegenerative disorders including AD and include several important considerations. In fact, changes in eating habits in persons with AD can be drastic, leading to multiple negative health outcomes. Aging may result in diminished taste perception leading compensatory changes in dietary intake, such as eating less or consuming more sweet and salty foods (Sergi et al., 2017). Taste disorders in the elderly are commonly overlooked or unrecognized but may predispose this population to a higher risk of developing diet-related diseases and malnutrition (Imoscopi et al., 2012). Physiological changes associated with aging, as well as many pharmaceuticals used for treating chronic diseases in the elderly are potential causes of taste disorders (Imoscopi et al., 2012). Even in the absence of taste alteration, eating habit changes are common across the dementia spectrum. In AD in particular, anorexia and appetite loss are most seen, along with preference for sweet foods (Valotassiou et al., 2021), including those with high sugar and carbohydrate content As noted, increased consumption of sugary and processed foods drives inflammation and insulin resistance. In addition to increasing the risk for AD development (Liu et al., 2022), the tendency for those diagnosed with AD to consume diets high in sugar and high glycemic index carbohydrates creates a scenario in which dysbiosis, immune dysregulation, and inflammation may persist in perpetuity without intervention.

Physical and mechanical functions further influence dietary intake. Oral health, including inadequate dentition is also closely related to malnutrition in the elderly (Nomura et al., 2019). Moreover, the brain regions that are affected in AD are also related to chewing function (mastication) (Campos et al., 2017). In one study of 16 persons with mild AD, masticatory function was correlated with cognitive status (Campos et al., 2017). Oral health, including dentition, are therefore vital factors to consider for both overall health and cognitive functioning.

In persons with AD, weight loss, often due to malnutrition, is a clinical feature observed in approximately 40% of patients and is associated with numerous adverse outcomes in an already difficult disease (Droogsma et al., 2013). Possible mechanisms include increased energy expenditure, alterations in nutrient uptake, and/or reduced food intake (Doorduijn et al., 2019). Notably, weight loss often precedes the onset of AD and older adults with poorer nutritional status demonstrate more pronounced cognitive decline (Doorduijn et al., 2023). In addition to malnutrition, loss of appetite and weight in persons with AD increases the risk of institutionalization and mortality (Valotassiou et al., 2021). Further nutritional concerns in persons with AD include sarcopenia and frailty (Unsal et al., 2023). In a recent study by Unsal et al. (2023), they aimed to evaluate the prevalence of nutritional disorders in a sample of 253 persons with AD. In their sample, they found that 64.8% had malnutrition or were at risk of malnutrition; 38.3% had sarcopenia; 19.8% were prefrail; and 80.2% were frail. Furthermore, they determined that malnutrition, frailty, and sarcopenia prevalence increased as AD stage progressed (Unsal et al., 2023). Another study by Ohta et al. (2019) analyzed physical functions associated with sarcopenia and frailty in participants with AD (*n* = 165) and MCI (*n* = 84) and cognitively intact controls. They found that cognitive and affective functions, activities of daily living, and physical functions worsened in MCI and AD in both genders, and were especially noticeable in females (Ohta et al., 2019). The extent to which gender differences in sarcopenia and frailty occur is still being explored. Certainly, these are major concerns that warrant proactive screening and intervention in both genders.

#### 1.2.2 Gender disparity in AD

Lifestyle choices, including diet, are an integral part of brain health. Overall, the literature consistently suggests that women tend to choose healthier foods (Gil et al., 2022; Jimenez-Morcillo and Clemente-Suárez, 2023; Feraco et al., 2024) yet suffer disproportionately from dementia, particularly Alzheimer’s disease (AD), the most common form (Derreberry and Holroyd, 2019), *and* remain underrepresented in the published literature (Ferretti et al., 2021). Of note, sex is one of the strongest risk factors for developing AD following age, as evidenced by the fact that two thirds of those clinically diagnosed with AD are women worldwide (Ferretti et al., 2021; Subramaniapillai et al., 2021), yet sex and gender have not yet been incorporated into precision medicine approaches (Nebel et al., 2018). In the United States alone, 4.1 million of the 6.7 million people aged 65 and older with AD are women (Alzheimer’s Association, 2023). Furthermore, women tend to live longer after diagnosis, but appear to deteriorate faster *and* suffer more severe cognitive deficits across multiple cognitive domains than men (Derreberry and Holroyd, 2019). Interestingly, males have a higher incidence of mild cognitive impairment (MCI), the often-prodromal stage of AD, but females are more likely to progress to AD (Mukhara et al., 2020). Men have also been found to have higher rates of comorbidities and mortality, and are more likely to exhibit sexually inappropriate behaviors (associated with the behavioral and psychological symptoms of dementia) requiring treatment than in women (Derreberry and Holroyd, 2019). Caregivers of persons with dementia are more likely to be women (Derreberry and Holroyd, 2019) and report more physical and mental health problems due to caregiver stress (Sheehan et al., 2021). Acute and chronic stress are correlated with poor dietary choices (Nitsch et al., 2021), both of which place the caregiver at increased risk for AD (Corrêa et al., 2019).

#### 1.2.3 Potential mechanisms of gender differences in AD

Several theories have been proposed to account for the more pronounced cognitive decline and worse disease course in women with AD. Genetic factors appear to be prominent, as possessing one or two apolipoprotein E ε4 alleles places women at higher risk for developing AD compared to men (Subramaniapillai et al., 2021). Notably, the brain and cognition are modulated by several external (e.g., environment) and internal (psychological) factors that interact with unique differences in sex/gender physiology, including menstrual cycles, hormone levels (Jäncke, 2018) and metabolism. Hormones levels, including sex hormones, can be affected by dietary habits across the lifespan and carry implications in a variety of sex-related and sex hormone-related diseases (Hirko et al., 2016; Insenser et al., 2018; Childs, 2020). Furthermore, women may be more negatively affected by certain risk factors, such as having the aforementioned apolipoprotein E ε4 allele and lower lifetime exposure to ovarian hormones (Subramaniapillai et al., 2021). The relationship between reproductive aging and cognitive aging is still in its infancy and the correlation, if any, is still being explored, but we do know that sex hormones play a pivotal role in cognition across the lifespan in both sexes (Ferretti et al., 2021), and these hormones are affected by dietary intake (Hirko et al., 2016), and may contribute to age-related cognitive decline (Gurvich et al., 2020). For example, a relatively consistent link between estrogen depletion has been linked to cognitive decline (Gurvich et al., 2020). Additionally, both of the major estrogen receptors (Erα and Erβ) are expressed throughout the brain, including neurons and glia which are involved in the neuroinflammatory process (Ávalos et al., 2018). Considering the knowledge that AD pathology in the brain begins years before the onset of symptoms, the pathogenic process may occur during the reproductive stage in women, before the cessation of ovarian function which further affects neural networks (Saha et al., 2024). Indeed, the literature supports the role of sex steroid hormones (i.e., estrogen, progesterone, testosterone) in neuroprotection in several neurological and psychiatric diseases, including cognitive decline (Mukhara et al., 2020). One mechanism by which estrogen exerts an anti-inflammatory response is its ability to downregulate matrix metalloprotease-9 (MMP-9) expression which thereby inhibits production of amyloid beta (Sinyor et al., 2020). However, the use of hormone therapy to prevent or treat cognitive decline remains controversial and inconsistent, owing to the complexity of hormone metabolism in the body (Gurvich et al., 2020).

AD pathology may manifest differently in the brains of males and females. Autopsy studies have demonstrated that women have more neurofibrillary tangles in the hippocampus, but not amyloid beta plaques, than men, suggesting a possible anatomic susceptibility within the brain (Ferretti et al., 2021). MRI studies also demonstrate higher rates of hippocampal atrophy in women with AD (Mukhara et al., 2020). This may help explain the more pronounced clinical symptoms in women with AD, as evidence suggest that women are more likely to express clinical symptoms despite the same amount of AD pathology (Ferretti et al., 2021). Being cognizant of potential anatomic vulnerabilities between genders may offer additional clinical insight into the broader context of guiding patients in best practices of dietary habits for brain health, which may be more gender-specific than previously thought. If female neuroanatomy is more susceptible to metabolic disturbances and inflammation, it is possible that females may necessitate more aggressive attention to anti-inflammatory dietary practices with an emphasis on nutrients known to support brain health.

### 1.3 Gender considerations of the microbiome

Research over the past few decades has started to uncover a critical link between the microbiome and human health and disease. Formed in early life, the gut microbiome is home to trillions of microbes consisting of bacteria, fungi, archaea, viruses, and phages that are influenced by genetic and epigenetic factors across the lifespan, including those that affect brain health (Walker et al., 2017). These microbes facilitate optimal brain health and function by modulating the integrity of intestinal and brain barriers and regulating homeostasis within the metabolic, immune, endocrine, and nervous systems (Chok et al., 2021). An imbalance of microbial diversity, or dysbiosis, can affect brain health via the microbiota-gut-brain axis (MGBA) through the secretion of toxins, cytokines, and short-chain fatty acids which in turn, affect gut permeability, immune response, and blood brain barrier integrity (Cammann et al., 2023). Growing evidence indicates that bacteria in the gut can release lipopolysaccharide (LPS) and amyloids that trigger microglial activation in the brain, which promote upregulation of proinflammatory cytokines and the culmination of neuroinflammation related to AD pathogenesis (Cammann et al., 2023). Due to the bidirectional nature of the MGBA, directly via the vagus nerve and indirectly via metabolites, inflammation in the periphery can translate to the CNS and contrarywise (Jamar et al., 2021).

As both estrogen and testosterone are both present and necessary in both genders, dysregulation of either can lead to a variety of pathologies, including cognitive decline. The “estrobolome” includes the gene repertoire of microbiota that are capable of metabolizing and modulating estrogens, and in parallel, the microbiota that synthesize testosterone constitute the “testrobolome” (Baker et al., 2017). Furthermore, estrogen serves a multifaceted role in human physiology and pathophysiology and serves as a mediator of intestinal inflammation (Jacenik et al., 2019), which itself can lead to neuroinflammation. Some of estrogen’s functions are also sex-dependent, such as its effect on GI motility that appears to be more pronounced in females than males (Jiang et al., 2019). This relationship is also bidirectional, as one of the principal regulators of circulating estrogens is the microbiome (Baker et al., 2017). Dysbiosis in the overall microbiome therefore affects, and is affected by, the estrobolome and may be an important driving force in cognitive decline (Baker et al., 2017), as well as a potential contributor to increased susceptibility among women.

### 1.4 Crossroads of gender, diet, inflammation, and neuroinflammation

Mounting evidence suggests that diet, inflammation, and neuroinflammation are inextricably connected and mediated to a large degree through the MGBA (Estrada and Contreras, 2019). Ultra-processed foods are a consistent descriptor of “unhealthy” foods in the literature (Elizabeth et al., 2020). Ultra-processed foods that laden with added sugar, saturated fat and trans-fat, high in sodium (Elizabeth et al., 2020), all of which tend to be consistent with the Western diet are known to cause disruptions in the microbiome that lead to an inflammatory cascade systemically and within the CNS, thereby raising the risk for AD development (Jena et al., 2018; Gonzalez Olmo et al., 2021; Więckowska-Gacek et al., 2021). Western-type diets promote an inflammatory immune response via upregulation of CRP, TNF-α, IL-1β, IL-6, and insulin, and have been associated with hippocampal damage, one of the key targets in AD; while the opposite effect is seen with high fiber diets (Kurowska et al., 2023). Chronic inflammation, or inflammageing, is highly deleterious and associated with a multitude of disease states (Ferrucci and Fabbri, 2018). Inflammageing increases an individual’s susceptibility to chronic morbidity, disability, frailty, and premature death (Ferrucci and Fabbri, 2018). As an important modifiable risk factor for a myriad of pathologies, diet can either induce or mitigate inflammation (Ferrucci and Fabbri, 2018) via the microbiome. Given the relationship between dysbiosis and exaggerated neuroinflammation, the Western diet has been implicated in neuropathologies such as memory impairment, neurodgenerative disorders (including AD), and depression (Gonzalez Olmo et al., 2021).

While “unhealthy” foods are ubiquitously unhealthy, the extent to which these deleterious effects occur between genders is unclear but an important consideration. Indeed, consumption of different types of foods may affect genders differently. For example, ultra-processed foods, such as fast foods, have been associated with depression (another condition involving neuroinflammation) (Gómez-Donoso et al., 2020; Lee and Allen, 2021; Bayes et al., 2022), yet this relationship may be more pronounced in women. Importantly, depression is both a risk factor for (Steenland et al., 2012; Edwards et al., 2019) and associated with Alzheimer’s disease as a neuropsychiatric symptom (Edwards et al., 2019; Banning et al., 2021).

The neurochemical milieu in the brain is also affected by diet. As the most abundant neurotrophin, brain-derived neurotrophic factor (BDNF) is one factor that mediates synaptic plasticity, low levels of which are associated with neurological disorders such has AD, yet lifestyle interventions such as diet and exercise can positively up-regulate BDNF, regulate insulin metabolism in the brain, and reduce inflammation (Xue et al., 2022). The decline of BDNF is associated with aging, and is more noticeable in females, the elderly, and obesity (Xue et al., 2022). Glud et al. (2019) conducted a 12-week randomized controlled trial to compare BDNF concentrations in 50 overweight or obese males and females after a weight loss intervention, including an exercise only group, diet only group, and diet plus exercise group. Their results demonstrated sex differences in response to the intervention. Specifically, the circulating BDNF was significantly changed by diet alone or combined with exercise only in women, but only by exercise in men. BDNF and lifestyle approaches to promote healthy levels of BDNF may be key therapeutic targets in AD, but responses to interventions may differ between genders.

As mentioned previously, changes in sex hormones levels are associated with aging and cognitive decline which are associated with AD. Diet and gastrointestinal function serve pivotal roles in hormone metabolism. Additionally, dietary intake of phytoestrogens such as genistein, which bind to estrogen receptors, can mimic estrogenic effects to affect brain function (Alwerdt et al., 2019). To investigate gender differences in the relationship between dietary phytoestrogens and cognition, Alwerdt et al. (2019) measured urinary genistein and speed of processing in 354 participants aged 65–85 years. They found that higher levels of genistein were linked to better speed of processing in women, but worse speed of processing performance in men, underscoring the importance of gender differences in dietary, supplemental, and pharmacological interventions (Alwerdt et al., 2019). It has also been purported that intermittent fasting, via the modulation of steroid hormones, can impact inflammatory processes that contribute to neurocognitive decline (Vasconcelos et al., 2016). Further, some evidence has suggested a potential therapeutic benefit of dietary restriction in reducing neuroinflammation (Bok et al., 2019), but the quality of foods remains a critical and uncompromising factor in health. The quality of an individual’s overall diet is a salient theme in this regard.

High fat diets are associated with diet-induced obesity, metabolic syndrome, and type-2 diabetes (Ávalos et al., 2018), which are further modifiable risk factors for AD (Edwards et al., 2019). High fat diets activate the hypothalamus to upregulate astrocytes and microglia, which appear to have a reciprocal relationship with obesity and metabolic dysfunction (Ávalos et al., 2018). High fat diets drive cytokine production from the hypothalamus that create numerous outward and downstream effects, including neuronal stress, glial activation, leptin resistance, and metabolic dysfunction (Ávalos et al., 2018), and due to the aforementioned bidirectional communication in the MGBA, inflammation in the brain and periphery often coexist. Furthermore, “unhealthy” diets can also drive changes in the microbiome that foment inflammation. For example, high fat and high sugar diets alter microbial composition in distinct patterns that lead to neuroadaptations (Jamar et al., 2021). Hyperlipidic diets have been shown to stimulate the Toll-like receptor 4 (TLR4) inflammatory pathway by increased LPS in the brain; overconsumption of sucrose is particularly detrimental to metabolic pathways, whereas fructose is especially detrimental to gut-barrier function, all of which contribute to inflammation, adiposity, and sugar addiction (Jamar et al., 2021).

### 1.5 Potential therapeutic interventions

Dietary and nutraceutical treatment interventions involving the gut microbiome and brain health are an emerging area of research to address one of the pathogenic processes in AD. Interventions that address the dysbiosis that results from poor dietary habits may halt, or at least dampen, the immune and inflammatory responses that contribute to AD development. These interventions include probiotics (exogenous supplementation of beneficial microbes), prebiotics (nondigestible carbohydrates that facilitate growth of beneficial microbes), synbiotics (a combination of pre-and probiotics), and fecal microbiota transplants, and various dietary approaches (Chok et al., 2021).

The CNS is a highly energy demanding system, with cognition and behavior directly related to the nutritional status of the organism (Gentile et al., 2020). The brain consists of mostly lipids, including phospholipids, sphingolipids, and cholesterol that maintain structure and function (Sun et al., 2018). Due to their diverse roles in brain cell function throughout the lifespan, dietary and supplementary phospholipids (e.g., phosphatidylcholine, phosphatidylserine, phosphatidylethanolamine, phosphoinositides, etc.) and omega-3 fatty acids (DHA, EPA, and ALA) have gained interest in the treatment of neurological disorders such as AD, but most studies that show support are animal models (Sun et al., 2018). While promising, more robust evidence in human trials is needed to establish supplemental guidelines in adults, but dietary sources of many nutrients help to maintain neuronal integrity and promote proper brain structure and function, as well as neurotransmitter synthesis synaptic transmission. For example, omega-3 fatty acids (found in foods such as fish, extra virgin olive oil, walnuts, and soybeans), are essential to neuronal membrane composition, fluidity, and function, and are important for synaptic plasticity, hippocampal neurogenesis, and learning (Gentile et al., 2020). Flavonoids increase hippocampal BDNF to maintain spatial working memory and promote hippocampal neurogenesis (Gentile et al., 2020). Additionally, several other individual nutrients and micronutrients have been studied for their role in the prevention and/or treatment of AD, including but not limited to antioxidants (Sinyor et al., 2020); anthocyanins (Khan et al., 2020); vitamin D (Kouba et al., 2023); vitamins C. E, B2, B6, B12, magnesium, and glutathione (Holton, 2021); zinc (Liu et al., 2023); phenolic compounds (Ciz et al., 2020), and many more. These nutrients, however, carry numerous roles in the body and the inconsistency in the literature, along with regulatory concerns in the supplement industry lend a great deal of warranted caution to clinicians and consumers, but can be appropriate especially in cases of deficiency. Whole foods are the preferable source of dietary nutrients when possible, and diets shown to be neuroprotective (e.g., Mediterranean, DASH, MIND, ketogenic) are rich in these anti-inflammatory elements to facilitate neuroprotection (Kurowska et al., 2023). While many mechanistic studies have focused on single nutrients, the synergistic effects of whole foods when consumed together as dietary patterns are likely to possess more influence on inflammatory processes and neurodegeneration (McGrattan et al., 2019).

Whole food sources, especially whole plant foods, confer numerous health benefit due to their high contents of functional macromolecules (e.g., polysaccharides, polyphenols, bioactive peptides, etc.), the molecular signally pathways of which modulate neuroinflammation (Huang et al., 2023). Whole foods rich in soluble dietary fiber (e.g., fruits, vegetables, grains, legumes, nuts) are also prebiotic sources (e.g., fructans and galacto-oligosaccharides) that feed the beneficial bacteria in the gut microbiome (Freijy et al., 2023). In a recent study comparing a high prebiotic diet versus probiotic supplementation versus synbiotics on mental health outcomes, the high prebiotic dietary intervention outperformed the probiotic supplementation (Freijy et al., 2023). Whole foods containing pre and/or probiotics may be more beneficial than supplementation with single strains in this regard. Bearing in mind the importance of one’s overall diet and the thousands of active components present in whole food sources (the average fruit or vegetable is estimated to contain over 15,000 different compounds from over100 chemical classes) (LeVatte et al., 2022), this section will focus on dietary approaches.

To date, the most widely studied dietary patterns to prevent and delay AD and related dementias (ADRD) are the Mediterranean (described earlier), DASH (Dietary Approaches to Stop Hypertension), MIND (Mediterranean-DASH Intervention for Neurodegenerative Delay), ketogenic diets, and modified Mediterranean-ketogenic diets (Ellouze et al., 2023). The DASH diet is similar to the Mediterranean diet but also emphasizes dairy consumption and limited intake of sodium, industrial sweets, and saturated fat (Pistollato et al., 2018), whereas the ketogenic diet emphasizes high intake of fats and low intake of carbohydrates. A systematic review by Van den brink et al. (2019) demonstrated that the Mediterranean, DASH, and MIND diets have been associated with less cognitive decline and a lower risk of AD, the strongest associations of which were observed in the MIND diet (Van den brink et al., 2019). Most predominantly, whole food plant-based dietary patterns have the most robust and consistent evidence in preventing and reducing the risk of ADRD (Ellouze et al., 2023). Overall, multi-component healthy dietary patterns appear to be more effective than single nutrient supplementation strategies for brain health management and reduced AD risk (Pistollato et al., 2018).

The MIND diet is a combination of the Mediterranean diet and the DAHS diet created specifically to reduce the risk of cognitive decline (Stefaniak et al., 2022). It emphasizes plant-based foods similar to the Mediterranean diet with an added emphasis on green leafy vegetables, nuts, and berries due to their known neuroprotective effects, as well as whole grains, seafood, poultry, olive oil, red wine, and limited intake of animal and high-saturated-fat foods (Stefaniak et al., 2022; Vu et al., 2022). It is based on the most compelling evidence in the neuronutrition field (Vu et al., 2022), accounting for the recent abundance of literature supporting its beneficial effects on cognitive function (Stefaniak et al., 2022) and reduced incidence of AD (Morris et al., 2015). The MIND diet has been found to be associated with better cognitive functioning independent of brain pathology (including AD), underscoring its impact on cognitive resilience (Dhana et al., 2021). Moreover, evidence supports the efficacy of the MIND diet in reducing the risk of cognitive impairment and dementia in populations outside of the US that are younger and culturally distinct (Krueger et al., 2022).

A systematic review by Devranis et al. (2023) compared the Mediterranean diet, MIND diet, and ketogenic diet in populations with cognitive decline. They found that all diets were efficacious in slowing cognitive decline. The Mediterranean diet excelled at global cognitive improvement after 10 weeks, the ketogenic diet had a beneficial effect in patients with diabetes mellitus and improved verbal recognition, and the MIND diet demonstrated benefit to obese patients and improved working memory, verbal recognition, memory, and attention (Devranis et al., 2023). A recent exploratory review of AD prevention and diet was conducted by Bhuiyan et al. (2023) found the Mediterranean diet and MIND diet lowered AD risk from 53 to 35%; high adherence to the Mediterranean diet and DASH had a 54 and 39% lower risk of developing AD, respectively; that the omega-6, PUFA, found in nuts and fish, can play most roles in the clearance of Aβ; and Vitamin D inhibits induced fibrillar Aβ apoptosis. This study provides support for the role of diet in AD risk as well as specific nutritional components on AD biomarkers (Bhuiyan et al., 2023).

The ketogenic diet consists of a high amount of fat and medium-chain triglyceride intake with very low carbohydrate intake, which leads to ketone body production that fuels the brain the absence of glucose (Lilamand et al., 2020). Clinical trials of the ketogenic diet and AD are limited, but have shown some improvement in cognitive performance in elderly adults with MCI and A (Włodarek, 2021). Some animal and human trials have suggested the potential for improved global cognition, memory, and executive functioning in cognitive impairment (Lilamand et al., 2020). It’s important to note that the type of fat intake is significant, as high dietary intake of saturated fats and trans-fats is correlated with an increased risk of AD (Frausto et al., 2021; Valentin-Escalera et al., 2024).

While there is strong evidence supporting Mediterranean-type diets in reducing disease risk, there may be gender differences that mediate this effect. A recent cross-sectional study by Barrea et al. (2024) explored sex-related difference in adherence to the Mediterranean diet and found differences in adherence and food preferences. Females displayed significantly higher adherence to the Mediterranean diet and consumed significantly more vegetables, fruits, legumes, fish/seafood, nuts, and sofrito sauce and less olive oil, butter, cream margarine, red/processed meats, soda, red wine, and commercial sweets than males. Females also had lower high sensitivity C-reactive protein (hs-CRP), an inflammatory marker, than their male counterparts (Barrea et al., 2024). An earlier study by Gu et al. (2021) included 2,435 older participants (60 years of age and older) to examine the association between the Mediterranean diet and cognition across gender and racial/ethnic groups. They found significant associations in global cognition and immediate recall in non-Hispanic whites only, and increased animal fluency scores in men but not women. Their results suggest that positive associations of the Mediterranean diet with cognition may be mediated by race and gender (Gu et al., 2021). The Mediterranean and Okinawan diets also include regular consumption of phytoestrogens, which can mitigate neuroinflammation, and have been associated with improved cognition (Alwerdt et al., 2019). The contribution of the ApoE ε4 genotype, which places women at higher risk for AD than men (Subramaniapillai et al., 2021), to dietary patterns is unclear (Berendsen et al., 2018). Berendsen et al. (2018) conducted a prospective cohort study of 16,058 women from the Nurses’ Health Study evaluated the association between the long-term adherence to the MIND diet and cognitive function and cognitive decline, and the interaction of the ApoE ε4 genotype. They found that the MIND diet was associated with better verbal memory, irrespective of ApoE ε4 status over 6 years, but no association was found with cognitive decline (Berendsen et al., 2018).

## 2 Discussion

The sum of the literature supports a strong trend in gender differences in dietary habits. The conclusions of the literature, that women overall have a healthier diet, is most puzzling when considering the increased rate and severity of disease course in Alzheimer disease in women compared to men. This suggests that more subtle but critical sex differences occur in AD pathogenesis, including the metabolism of food and its influence on the gut microbiome, hormones, immune system, and CNS. Examined individually, these factors are well-established. Nutrition intake is related to AD risk; it influences the health of the microbiome; it contributes to hormone balance, immune tolerance, and brain health. Indeed, diet may serve as either a risk or protective factor for AD, but how and to what extent this differs between genders remains unknown. It is possible that at the epigenetic level, certain modifiable lifestyle factors interact differently between genders and may be modulated via the microbiome, which is a relatively recent area of research. A wholistic view of these multidirectional interactions is imperative to effect change and provide actionable advice to patients with gender-specificity as appropriate.

There are some areas of medical advice that are reasonably believed to be ubiquitously beneficial, such as a whole foods plant-based diet, regular exercise, adequate sleep, stress management, and social connection. Even in their ubiquity, these wise evidenced-based recommendations may interact with and affect genders differently. Owing to unique and subtle differences in genetics, metabolism, psychology, and environment, gender differences in disease pathomechanisms are an important consideration in medical care. It is the belief of this author that both genders should always be treated fairly, but not necessarily equally, as the plethora of epigenetic influences to which we are exposed interact quite differently in both sexes, as we are beginning to understand—including diet, pharmaceuticals, and stress. The interplay between hormones, dietary habits, and the inflammatory response has demonstrated several ways in which genders diverge in the pathogenesis of various disease, such as cardiovascular disease, metabolic syndrome, and neurodegenerative disorders. It is therefore incumbent upon medical providers to utilize this knowledge to inform patients of current evidence to create personalized care plans aimed to prevent, slow, and treat said diseases. Research in these areas is still young and in need of refinement, but the literature thus far has created an opportunity to update best practices in medical care. Nonetheless, there are many limitations to consider, including the bias inherent in self-report and accuracy of dietary recall. Limitations notwithstanding, it is clear that certain diseases carry a gender bias. The crossroads of sex hormones, the gut microbiome, immune activation, and inflammation are allowing for great strides in understanding important gender differences in Alzheimer’s disease and will hopefully translate into actionable medical advice, prevention, and treatment options in this vulnerable population.

## Author contributions

AW: Writing – original draft, Writing – review & editing.
